# High-performance renal imaging with a radiolabeled, non-excretable chimeric fusion protein

**DOI:** 10.7150/thno.66417

**Published:** 2021-09-03

**Authors:** Dawei Jiang, Muhsin H. Younis, Xiaoli Lan, Weibo Cai

**Affiliations:** 1 Department of Nuclear Medicine, Union Hospital, Tongji Medical College, Huazhong University of Science and Technology, Wuhan, 430022, China; 2 Departments of Radiology and Medical Physics, University of Wisconsin - Madison, Madison, WI, 53705, USA; 3 Hubei Key Laboratory of Molecular Imaging, Wuhan, 430022, China

**Keywords:** nuclear medicine, renal scan, recombinant protein, single photon emission computed tomography (SPECT), vascular endothelial growth factor (VEGF), polybasic tag (PBT), neonatal Fc receptor, Tc-99m

## Abstract

Ideal nuclear imaging tracers should exhibit high target uptake and low background signal. Traditional renal scintigraphy and SPECT scans examine kidney function *via* static or dynamic tracing of radioactive probes in the kidneys. The lack of tracer affinity to specific biological processes and high background uptake from urinary excretion have added many difficulties to precision renal diagnosis. In this issue of *Theranostics*, Jin and colleagues innovatively devised a recombinant probe for preferential kidney imaging through targeting of tubular neonatal Fc receptor and proximal tubular basement membrane for sustained tubular reabsorption and accumulation. This work has broad implications regarding how an in depth understanding of physiology and pathology may be of service for tracer development, renal diagnosis, and disease theranostics.

The fundamental limitations in nuclear imaging, of renal dysfunction and other diseases, is a lack of tracers with molecular precision for the disease to be probed 1-3. Radiochemistry leverages biological and chemical tools to formulate, manipulate, and regulate imaging tracers for nuclear medicine and molecular imaging. An ideal radiopharmaceutical calls for excellent stability, minimal toxicity, and, most importantly, exclusive specificity to its target. Good examples are radioiodine (^125^I, ^131^I) and ^99m^Tc-MDP (methylene diphosphonate) with their known avidity to the thyroid and bones 4, 5. Other tracers with preferential liver metabolism have also been explored to examine enterohepatic circulation and the gastrointestinal tract 6. These cases show that specific organ targeting plays a vital role in tissue imaging and organ function evaluation. However, things get a bit tricky when imaging the kidneys. Renal excretion comprises three major processes: glomerular filtration, tubular reabsorption, and tubular secretion 7. Blood tests, urine tests, and ultrasound imaging are routinely used in the initial kidney examination and treatment evaluation after therapeutic interventions 8. These methods are unable to fully depict kidney function quantitatively.

Radionuclide imaging tracers have been developed for renal diagnostic scans 9, employing tracers such as diethylenetriaminepentaacetic acid (DTPA), mercapto-acetyl-triglycine (MAG3), dimercaptosuccinic acid (DMSA), and glucoheptonate (GHA) 10-12. However, in current clinical practices, different SPECT tracers can only monitor each of the three aforementioned major renal processes. For example, ^99m^Tc-DTPA is mainly used to measure the dynamics of glomerular filtration in renal scintigraphy, while ^99m^Tc-MAG3 is common in measuring tubular excretion. ^99m^Tc-DMSA and ^99m^Tc-GHA are kidney-avid, but both present relatively high whole-body retention, making them suitable for static renal scintigraphy.

Additionally, passive tracer excretion *via* the kidneys may hinder active observation of renal dysfunction, scaling down necessary imaging contrast needed for confident diagnosis. Low molecular weight antibody fragments in the forms of Fab, single-chain Fv fragment (scFv), diabody, and nanobody, have all been found to excrete from the kidneys 1, 13. Going through lysosomal proteolysis, glomerular filtration, and subsequent tubular reabsorption, these radioactive renal metabolites display rapid renal excretion and steady kidney retention, adding difficulties to kidney function interpretation. As such, efforts have been taken to reduce renal tracer uptake. Previous studies have shown that PEGylation of nanobodies can decrease tracer excretion, thus extending their blood circulation 14. Besides, premedication with fructose or sodium maleate may reduce renal uptake of an anti-CD38 nanobody, [^68^Ga]Ga-NOTA-Nb1053 15. However, these strategies reduce kidney accumulation and urinary excretion of radioactive tracers but fail to help with kidney dysfunction diagnosis.

Imaging comprehensive renal function while avoiding non-specific urinary excretion has been a real challenge. The desired tracer should have a positive and stable kidney accumulation regulated by a known biological process. In cases of kidney injury, acute or chronic, the tracer needs to faithfully reflect biological changes of the kidney in the living organism.

In this issue of *Theranostics*, Jin and colleagues purposely combined the IgG fragment crystallizable (Fc) domain with the VEGFA extracellular matrix (ECM) binding domain to construct a chimeric fusion protein 16. The Fc segment of IgG1 binds to neonatal Fc receptors, responsible for recollecting filtered IgG and serum albumin from excretion and protecting the recombinant protein from lysosomal degradation. On this basis, a polybasic tag (PBT) from VEGFA targeting tubular base membrane matrix is added to maintain the tracer's stable retention in renal parenchyma. As expected, the as-formed low molecular weight compound first passes through glomerular filtration due to its small size, binds to tubular neonatal Fc receptors to break free from lysosomal catabolism, and targets proximal tubular base membrane matrix for reabsorption. After Tc-99m labeling and radiochemistry characterization, the obtained SPECT tracer successfully took part in glomerular filtration and tubular reabsorption while escaping lysosomal degradation and urinary clearance in mice and rats. On the contrary, the free Fc segment showed persistent blood circulation in animals.

While most nuclear imaging tracers are developed to probe a known target in diseased sites such as cancer 17-19, the central nervous system 20, 21, or other organs 1, this simple yet elegant design of a SPECT renal probe emphasizes how specific organ targeting guided by known biological processes can be of service for nuclear imaging. Similar to employing radioiodine for thyroid imaging, the combination of Fc domain for tubular recollection and PBT for kidney enrichment added a nice touch of finesse to the final probe, showcasing the authors' in depth understanding of kidney physiology and pathology. Pu and colleagues developed a series of molecular renal probes (MRPs) for optical imaging of kidney dysfunction 22. When going through renal clearance, MRPs will efficiently respond to early events of kidney injury such as oxidative stress, lysosomal degradation, and cellular apoptosis, providing optical readouts for early evaluation of renal impairment. Our group have also developed a series of DNA nanostructures for exclusive kidney imaging and acute kidney injury (AKI) intervention. Tetrahedral DNA nanostructures at sizes between 10-20 nm underwent specific urinary excretion, allowing for early diagnosis of unilateral ureter obstruction even at day 1 after animal model establishment 23. DNA origami nanostructures at larger sizes (> 100 nm) presented exclusive kidney retention for as long as 24 h. This enriched and prolonged renal accumulation was then harnessed for AKI alleviation in murine models 24.

This study is also an excellent example of the development of hybrid nuclear imaging tracers. The direct fusion of two targeting domains perfectly delineated kidney parenchyma for imaging of multiple renal diseases. Currently, nuclear imaging probes based on bispecific peptides or antibodies have piqued great interest for cancer diagnosis. Combining two tumor-targeting peptides may expand the diagnostic scope of nuclear imaging tracers 25. The conjugation of two F(ab) fragments yielded highly improved tumor uptake from below 10 percent injected dose per gram (%ID/g) to approximately 35 %ID/g 26, 27. Moreover, the direct expression of bispecific or even tri-specific antibodies using molecular biology methods may shift the diagnostic and therapeutic landscape of nuclear imaging 1, 28.

The work presented by Jin and colleagues in this issue of *Theranostics* showed the authors' clear understanding of kidney excretion and excellent execution of tracer development, which is highly desired in the pursuit of ideal theranostics for disease imaging and treatment. The employed methods may also have broad implications for the development of recombinant proteins specific for multiple targets to elicit integrated effects for nuclear imaging.
